# Association between blood concentration of sertraline and its effectiveness and safety in major depression: exploratory results of the PREDEP-SERT study

**DOI:** 10.1192/j.eurpsy.2026.11861

**Published:** 2026-07-31

**Authors:** P. Molero, C. Canga-Espina, B. Tapia-Alzuguren, F. Ortuño, A. Aldaz

**Affiliations:** 1 Department of Psychiatry and Clinical Psychology, Clínica Universidad de Navarra; 2 Instituto de Investigación Sanitaria de Navarra (IdiSNA); 3 Pharmacy Services, Clínica Universidad de Navarra, Pamplona, Spain

## Abstract

**Introduction:**

Antidepressant medication is an integral part of the treatment for major depressive disorder (MDD), and selective serotonin reuptake inhibitors (SSRIs) are common first line options. Genetic and environmental factors can lead to toxicity or lack of efficacy within the established dose ranges of SSRIs, requiring the use of lower or higher doses in order to avoid the risk of clinical complications due to toxicity or excessively prolonged response latency. The determination of blood levels by means of therapeutic drug monitoring (TDM) may be determinant in this regard. In the case of sertraline, further evidence is needed regarding the optimal therapeutic range of this drug in MDD.

**Objectives:**

This study aims to assess the optimum ranges of blood concentrations of sertraline associated with therapeutic response and safety, and relevant clinical, demographic and genetic factors.

**Methods:**

The PREDEP-SERT study (PREdictors of response in DEPression treated with SERTraline) is an ongoing, single-center, observational, longitudinal, ambispective cohort study of patients with MDD to investigate the association between blood concentration of sertraline and its effectiveness, tolerability and safety (pre-registered study in Spain, REec number: 0014-2022-OBS) (Molero et al. PLoS One 2025; 20(8): e0325335). It adopts wide inclusion and exclusion criteria to enroll patients that are representative of real-world clinical practice. It includes an exploratory retrospective subcohort and a subsequent 6-month follow-up prospective subcohort, with repeated measures (blood concentrations of sertraline and clinical outcomes) at scheduled timepoints up to 6 months. It is focused on relevant clinical, pharmacogenetic and sociodemographic potential predictors, confounders and effect modifiers.

**Results:**

The PREDEP-SERT study protocol will be presented, together with exploratory retrospective results including the effects of sex, age, weight, smoking and comedication, the possible role of CYP2B6, CYP2C19, and CYP2D6 isoforms and phenoconversion in sertraline pharmacokinetics, and a retrospective, exploratory determination of an optimal therapeutic range.

**Conclusions:**

If confirmed prospectively, these results may facilitate the treatment of moderate to severe depression, prove the clinical utility of TDM in certain clinical scenarios of major depression treated with sertraline and serve as a case in point for other SSRIs.

**Disclosure of Interest:**

P. Molero Grant / Research support from: research grants from the Ministry of Education (Spain), the Government of Navarra (Spain), the Spanish Foundation of Psychiatry and Mental Health and AstraZeneca. Principal investigator of several studies supported by Janssen and Novartis about the efficacy and safety of novel pharmacological treatments for depression., Consultant of: Clinical consultant for MedAvanteProPhase and Worldwide Clinical Trials Limited. Lecture honoraria from or has been a consultant for AB-Biotics, Adept Field Solutions, Dialectica, Guidepoint, Janssen, Novumed, Roland Berger, Scienta, and Survey Healthcare Global, received travel support for taking part in scientific meetings in the last 3 years (air/ground tickets + hotel) from Boston Scientific and Janssen, C. Canga-Espina : None Declared, B. Tapia-Alzuguren : None Declared, F. Ortuño: None Declared, A. Aldaz: None Declared

